# Topographical Evaluation of Neovessels in Proliferative Diabetic Retinopathy by Using a Multimodal Imaging Approach

**DOI:** 10.3390/jcm15176762

**Published:** 2026-08-31

**Authors:** Eliana Costanzo, Vito Fenicia, Sara Giammaria, Maria Sole Polito, Giulia Mecarelli, Monica Varano, Mariacristina Parravano

**Affiliations:** 1 IRCCS–Fondazione Bietti, Via Livenza, 3, 00198 Rome, Italy; sara.giammaria@fondazionebietti.it (S.G.); mariasole.polito@fondazionebietti.it (M.S.P.); giulia.mecarelli@fondazionebietti.it (G.M.); monica.varano@fondazionebietti.it (M.V.); mariacristina.parravano@fondazionebietti.it (M.P.); 2 UniCamillus-Saint Camillus International University of Health Sciences, Via Sant’Alessandro, 00131 Rome, Italy; vito.fen@gmail.com

**Keywords:** PDR, disc neovascularization, FAZ area, NVE, NVD, ischemic damage

## Abstract

**Background:** To evaluate structural and microvascular parameters in proliferative diabetic retinopathy (PDR) eyes, based on topographical location of neovessels (NVs). **Methods:** Retrospective cross-sectional study including PDR eyes without clinically significant macular edema. Patients were stratified on NVs location: disc (NVD), elsewhere (NVE), both (NVD + NVE). Optical coherence tomography (OCT) and OCT-angiography (OCTA) were performed to measure central macular thickness (CMT), choroidal thickness (ChT), retinal layer thicknesses, foveal avascular zone (FAZ) area, retinal perfusion density (PD) and choriocapillaris flow deficit (CC FD%). **Results:** Twenty-six PDR eyes from 26 patients (20 males, mean age 53.3 ± 14.2 years) were analyzed, 6 with NVD, 14 with NVE and 6 with NVD + NVE, respectively. No significant differences in CMT, ChT, PD and CC FD% between groups were found. OCT layer-by-layer analysis showed lower inner retinal layer thicknesses in NVE group. FAZ area was larger in NVD (*p* = 0.05). In presence of NVD, the regression analysis between FAZ and OCTA metrics showed a negative linear relationship with PD, and a positive relationship with CC FD%. **Conclusions:** In NVD group, a thickening of inner retinal layers and FAZ enlargement were found. These results support the hypothesis of a more pronounced ischemic damage in PDR eyes with NVs at the optic disc.

## 1. Introduction

Proliferative diabetic retinopathy (PDR) is the most advanced stage of diabetic retinopathy (DR) characterized by the development of retinal neovessels (NVs) located elsewhere (NVE), at the optic disc (NVD), at the angle (NVA) and/or at the iris (NVI) [1]. The pathophysiology of this condition is sustained by a microangiopathy characterized by capillary non perfusion, microaneurysms and retinal ischemia [2,3,4] and could potentially lead to several sight-threatening ocular complications as vitreous hemorrhages and tractional retinal detachment. It was also known that the prompt detection of NVD/NVE is essential for timely management and vision preservation [1]. However, several aspects regarding the influence of topographical location of NVs and the general condition of the patient remain poorly defined. This relationship likely reflects varying degrees of severity of the underlying diabetes, as well as the need for tailored managmenet approach.

The standard for the diagnosis of PDR is the fundus examination and, with the use of fluorescein angiography (FA), it was also possible to differentiate the NVs from other microvascular abnormalities, also detecting more peripheral lesions throughout the ultra-wide field imaging [5,6]. Extending the field of view, it was possible to evaluate that NVs are asymmetrically distributed throughout the entire retina, and in presence of NVD, a higher capillary non-perfusion (CNP) in all four quadrant of the retina were found compared to those without, indicating a more advanced stage of PDR [1,7]. Furthermore, recent evidences reported more extensive non-perfusion areas (NPAs) as well as a reduction of vessel density (VD) measured by optical coherence tomography angiography (OCTA) were found in eyes with NVD compared to those without [1,7,8]. On the other hand, further data suggested that the NVs location is associated with different functional outcomes, where patients exhibiting NVD have poorer visual prognosis and are more resistant to anti-VEGF therapy than those with NVE [9].

It was unclear which processes could drive the development of NVs in different locations and poor data are available to understand if different retinal NVs’ types (NVD vs. NVE) might be associated with different vascular profile of DR.

In this scenario, multimodal imaging offers a valuable non-invasive tool to explore retinal structural layers while assessing microvascular impairment, supporting clinical decisions on detailed disease characterization and timely management. OCTA integrates findings from other invasive imaging techniques, allowing a quantitative evaluation of macular status and correlating this information with the status of the peripheral retina.

The aim of this pilot study was to explore microvascular and structural macular parameters of PDR distinguishing between NVD and NVE by using a multimodal imaging approach.

## 2. Materials and Methods

### 2.1. Study Design and Participants

This retrospective cross-sectional observational study was performed at IRCCS-Fondazione Bietti of Rome, Italy. The study adhered to the tenets of the Declaration of Helsinki and was approved by the Institutional Review Board of the IRCCS-Fondazione Bietti (Comitato Etico Centrale I.R.C.C.S. LAZIO Sezione IRCCS I.F.O.–Fondazione G.B. Bietti-N.114/21/FB).

Two experienced examiners (EC and MP) identified the eyes with PDR based on the analysis of color fundus photographs, according to the modified Early Treatment Diabetic Retinopathy Study (ETDRS) retinopathy severity scale [10] and on the FA images.

All patients received a multimodal imaging assessment consisting of UWF-FA, optical coherence tomography (OCT) and OCTA, as part of the standard clinical approach.

Inclusion criteria were: patients > 18 years with type 1 or type 2 diabetes (T1DM or T2DM), any ethnicity, active PDR without clinically significant-macular edema, treatment-naïve or previously treated PDR, a complete imaging procedure.

Exclusion criteria were: laser photocoagulation in the macular region, previous retinal surgery or intravitreal injection therapy in the prior 6 months, any maculopathy caused by other than DR, significant lens opacity and refractive error >−6 diopters spherical equivalent (SE) or >+4 diopters SE. Poor quality images with a signal strength index (SSI) lower than 6 for the PLEX^®^Elite OCTA (Carl Zeiss Meditec, Inc., Dublin, CA, USA) or with significant motion artifacts (seen as large dark or grey lines on the enface angiograms) were also excluded to the analysis.

### 2.2. Study Imaging Protocol

All patients underwent the following imaging procedures in the same session day:

- Ultra-wide field fluorescein angiography (UWF-FA) by using Optos Advance V5.1.11.117461 (Optos, PLC, Dunfermline, Scotland, UK). The ultra-wide-angle Optos Advance was used to acquire 200° standard fovea-centered FA images in mydriasis, after dye injection of fluorescein, followed by superior, inferior, nasal, and temporal images obtained using the device’s fixation light in the early, intermediate and late phases of angiogram, as per clinical practice, to evaluate the neovessels’ location and activity [11].
- Spectral Domain (SD-)OCT by using Spectralis (Heidelberg Spectralis, Heidelberg Engineering, Heidelberg, Germany) with the OCT volume scan performed on a 20 × 20° cube, which consisted of 49 horizontal B-scans with 20 averaged frames per B-scan centered over the fovea. All B-scan images were checked for errors in automatic segmentation, and a manual correction was made in case of segmentation errors [12].
- Swept Source (SS-)OCTA images by using the PlexElite 9000 device (Carl Zeiss Meditec, Inc., Dublin, CA, USA). This device uses a swept laser source with a central wavelength of 1050 nm and a bandwidth of 100 nm, with an axial resolution of approximately 5 μm and a lateral resolution estimated at approximately 14 μm [13]. OCTA images were acquired using a 6 × 6 mm volume captured with FastTrac eye motion correction software (version 1.7.027959, Carl Zeiss Meditec, Inc., Dublin, CA, USA). The built-in segmentation software automatically segmented the whole retina slab, the superficial capillary plexus (SCP), the deep capillary plexus (DCP) and the choriocapillaris (CC) slab; the whole retina vasculature slab includes automatic segmentation from the inner limiting membrane (ILM) up to 70 µm above the retinal pigment epithelium (RPE) [14]; the SCP was segmented between the ILM and the inner plexiform layer (IPL); for the DCP, the upper limit was the IPL and the lower was defined by the outer plexiform layer (OPL); for the CC the image was segmented using a 20 µm thick slab, placed 29 µm under the RPE fit boundary, that corresponds to Bruch’s membrane [15]. The correctness of retinal boundaries segmentation was checked by two experienced examiners and manually adjusted in case of misplacing.

### 2.3. SD-OCT Parameters

The quantitative OCT parameters were: (i) central macular thickness (CMT), automatically measured using instrument software on the macula map through an inbuilt software of Heidelberg Spectralis, (ii) choroidal thickness (ChT), manually measured by using a caliper integrated into the device as the distance between hyperreflective inferior limit of RPE and the hyperreflective sclera-choroidal junction, in a line passing through the fovea [16] (iii) thicknesses of all retinal layers (see below). To measure the thickness of retinal layers, the OCT volume scan centered over the fovea was analyzed by using the software Heidelberg Eye Explorer (version1.10.2.0) to perform individual retinal layer segmentation, while the automated segmentation lines were examined to check the proper segmentation. Individual retinal layers were defined as follows [17]: retinal nerve fiber layer (RNFL; distance between the inner limiting membrane and outer edge of GCL), ganglion cell layer (GCL; distance between outer edge of RNFL and outer edge of GCL), inner plexiform layer (IPL; distance between outer edge of GCL and outer edge of IPL), inner nuclear layer (INL; distance between outer edge of IPL and outer edge of INL), outer plexiform layer (OPL; distance between outer edge of INL and outer edge of OPL), and outer nuclear layer (ONL; distance between outer edge of OPL and external limiting membrane). The thickness of individual layers was measured in the central, parafoveal and perifoveal subfield ring of the ETDRS grid, which was classified as a central 1 mm, parafoveal 3 mm and perifoveal 6 mm rings; the parafoveal and perifoveal ring values was calculated as the mean of inferior, nasal, superior and temporal subfield thicknesses.

### 2.4. SS-OCTA Parameters and Analysis

The quantitative OCTA parameters, evaluated on the 6 × 6 mm images, were: (i) foveal avascular zone (FAZ) area, (ii) perfusion density (PD) at the whole retina vasculature, SCP and DCP and (iii) the flow deficit of the CC slab (CC FD).

The quantitative OCTA parameters were measured as follows: the OCTA whole retina vasculature slab was opened on FIJI (an expanded version of ImageJ: 2.0.0-rc-69/1.52p; National Institutes of Health) [18], the FAZ border was manually out-lined, and the surface area, expressed in mm^2^, was measured as previously reported [19]. For the quantitative evaluation of FAZ the measurement through the OCTA was superior compared to FA in PDR patients [20]. In addition, the slabs were binarized using Mean thresholding algorithm to generate a black and white image for measuring the PD [19,21]. The PD defines the ratio of the area occupied by the vessels divided by the total area, providing complete vasculature information in terms of size and length [21]. Furthermore, the CC FD was calculated, expressed as percentage (CC FD%). The obtained CC images were imported in Fiji ImageJ (software version 2.0.0, National Institute of Health, Bethesda, MD, USA; available at http://rsb.info.nih.gov/ij/index.html (accessed on 25 February 2025)) and each CC image was compensated adjusting for shadowing artifacts and removing retinal vessel projection artifacts [22] and then binarized using the Phansalkar method (with a window radius of 3 pixels) [23,24,25]. In order to quantify the CC flow, the “analyze particles” command was employed. The FD represents the absence of flow or flow deficits appreciable in the CC slab.

### 2.5. Study Groups

**Figure 1:**
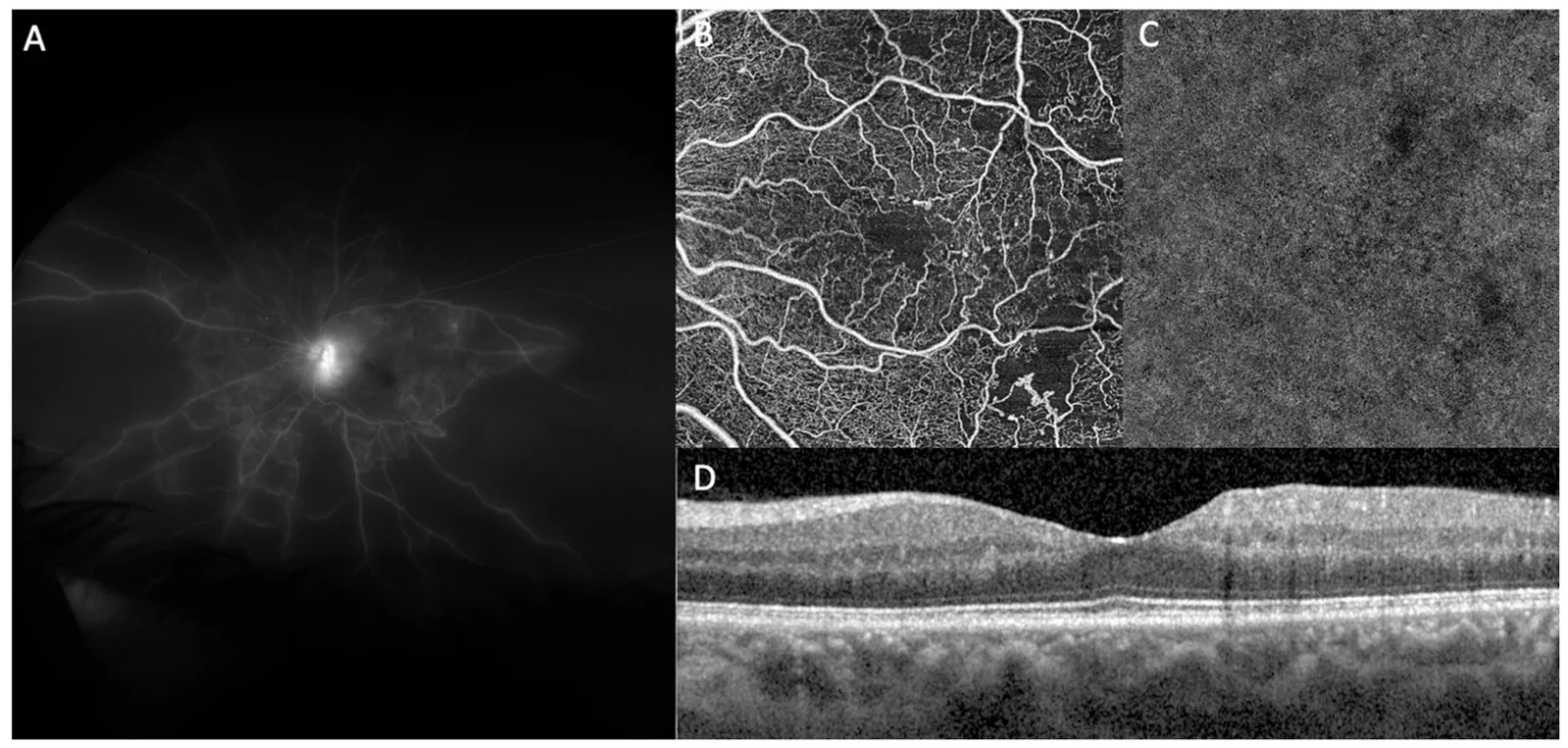
PDR eye with NVD. (**A**): Fluorescein angiogram of PDR eye with disc neovessel; (**B**): 6 × 6 OCTA slab of the whole retina; (**C**): 6 × 6 OCTA slab of the choriocapillaris; (**D**): SD-OCT line passing through the fovea.

**Figure 2:**
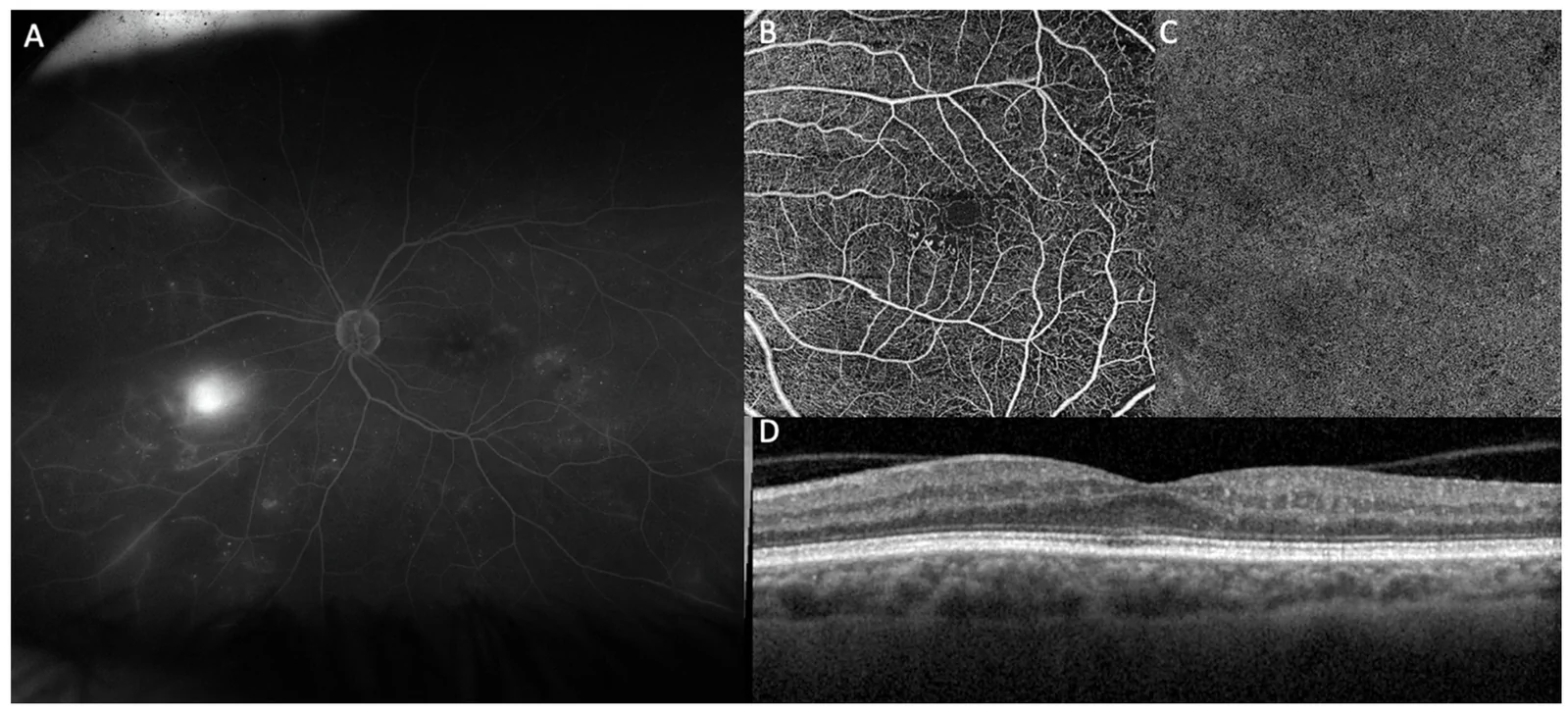
PDR eye with NVE. (**A**): Fluorescein angiogram of PDR eye with neovessel elsewhere in the mid nasal peripheral retina; (**B**): 6 × 6 OCTA slab of the whole retina; (**C**): 6 × 6 OCTA slab of the choriocapillaris; (**D**): SD-OCT line passing through the fovea.

**Figure 3:**
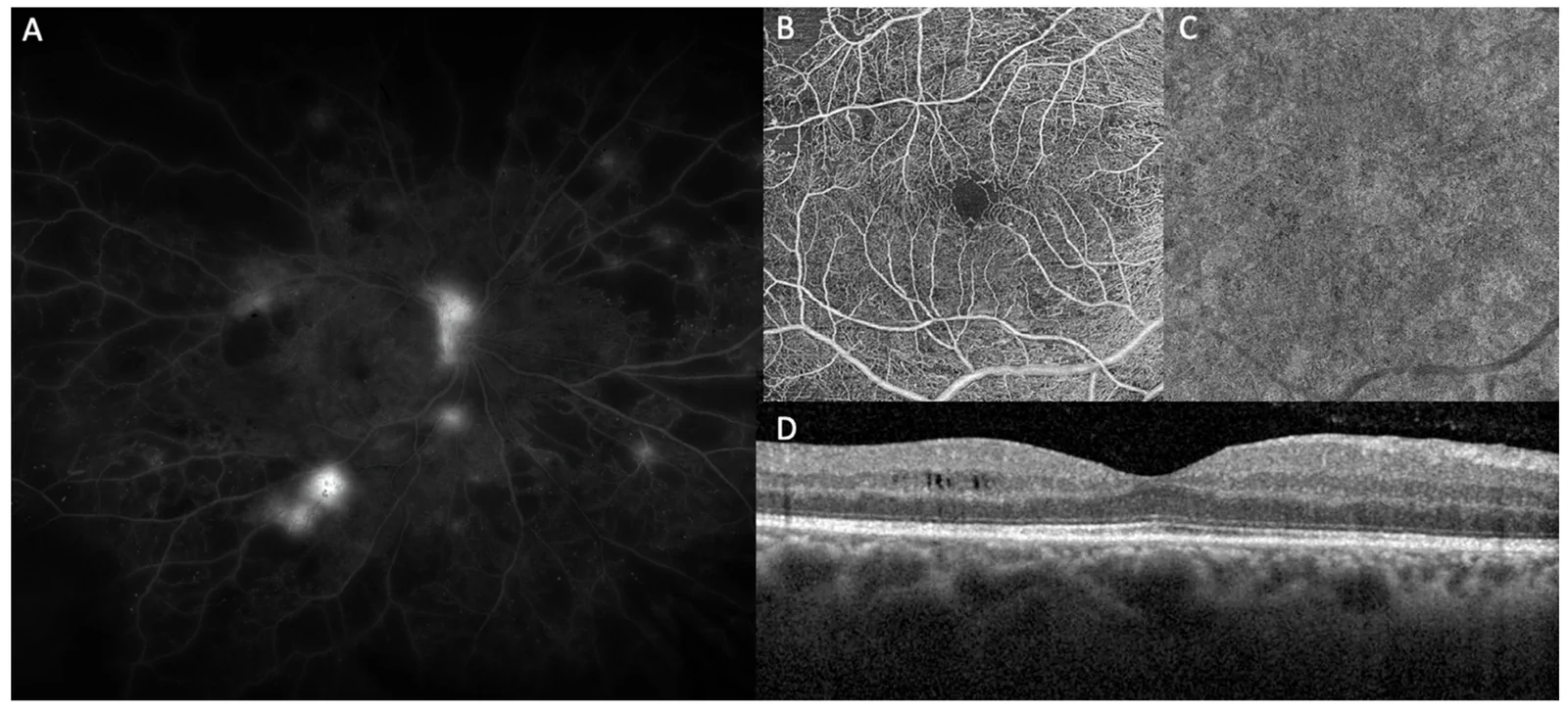
PDR eye with NVD + NVE. (**A**): Fluorescein angiogram of PDR eye with disc neovessel and in the mid nasal peripheral retina; (**B**): 6 × 6 OCTA slab of the whole retina; (**C**): 6 × 6 OCTA slab of the choriocapillaris; (**D**): SD-OCT line passing through the fovea.

The patients were subdivided in three groups based on the NVs location: (i) disc (NVD), (ii) elsewhere (NVE), (iii) disc + elsewhere (NVD + NVE) by using the UWF-FA frames to correctly analyze the images [1]. Figure 1, Figure 2 and Figure 3 showed representative examples of multimodal imaging of NVD, NVE and NVD + NVE, respectively.

### 2.6. Statistical Analysis

Demographic and ocular characteristics of patients included were summarized with mean and standard deviation (SD) if continuous variables or frequency (%) if categorical. After assessing normal distribution of continuous variables with the Shapiro-Wilk test, Kruskal-Wallis test was used to compare OCT and OCTA parameters between study groups and Dunn-test with Bonferroni correction for multiple comparisons was used for post-hoc analysis. Linear regression was used to explore the relationship between OCTA parameters. *p* value < 0.05 was considered statistically significant.

All statistical tests were performed using open-source software R (version 4.5.1) (R Core Team (2023). _R: A Language and Environment for Statistical Computing_. R Foundation for Statistical Computing, Vienna, Austria).

## 3. Results

Twenty-six eyes from 26 patients affected by PDR (20 males and 6 females, 16 T2DM and 10 T1DM, 21 Caucasic and 5 from Asiatic countries, mean age 53.3 ± 14.2 years) were included in the analysis. 17 out of 26 eyes were treatment-naïve while 9 eyes have previously received peripheral retinal photocoagulation, no patients received macular laser treatments or previous anti-VEGF intravitreal injections.

The mean ± SD CMT and ChT values in the overall population were 311 ± 39.4 μm and 288.4 ± 68.4 μm, respectively. The mean ± SD values for OCTA parameters in the overall population were: FAZ area 0.41 ± 0.22 mm^2^, PD in the whole retina 0.43 ± 0.02, PD in the SCP 0.42 ± 0.03, PD in the DCP 0.41 ± 0.03, CC FD% 31.66 ± 8.9.

Based on the NVs location, the patients were stratified as follows: 6 NVD, 14 NVE and 6 both types of NVs (NVD + NVE).

The NVD group consisted of 6 patients: 4 males and 2 females, 2 T2DM and 4 T1DM, 3 Caucasic and 3 from Asiatic countries, mean age 45.2 ± 15.2.

The NVE group consisted of 14 patients: 12 males and 2 females, 10 T2DM and 4 T1DM, 12 Caucasic and 2 from Asiatic countries, mean age 55.5 ± 14.

The NVD + NVE group consisted of 6 patients: 4 males and 2 females, 4 T2DM and 2 T1DM, all Caucasic, mean age 56.3 ± 12.8.

The Table 1 reports the OCT and the OCTA data of overall population and stratified by groups (NVD vs. NVE vs. NVD + NVE).

### 3.1. OCT Results

The analysis of CMT and ChT showed no significant differences between groups, *p* = 0.09 and *p* = 0.66 respectively.

The analysis of all retinal layers divided by ETDRS rings (1 mm foveal, 3 mm parafoveal and 6 mm perifoveal) showed specifically significant differences between groups. In particular, in the 6 mm perifoveal ring, significant differences in RNFL, GCL, and IPL thicknesses between groups were found (*p* = 0.01 for RNFL and GCL; *p* = 0.02 for IPL), where the NVE group exhibited a lower thickness of these layers followed by NVD group and then by NVD + NVE group. In the parafoveal ring only IPL thickness resulted statistically significant different between groups (*p* = 0.02) with the same trend of perifoveal ring. No differences for these layers in the foveal ETDRS ring were found.

Thickness values of INL, OPL and ONL were not statistically significant different between groups in all ETDRS rings.

### 3.2. OCTA Results

FAZ area was 0.55 ± 0.18 mm^2^ in NVD group, 0.34 ± 0.16 mm^2^ in NVE group and 0.42 ± 0.32 mm^2^ in NVD + NVE group (*p* = 0.051).

Other OCTA metrics, PD in whole retina, PD in SCP, PD in DCP and CC FD% showed no significant differences between groups (all *p* > 0.05). All data were reported in Table 1.

### 3.3. Regression Analysis

**Figure 4:**
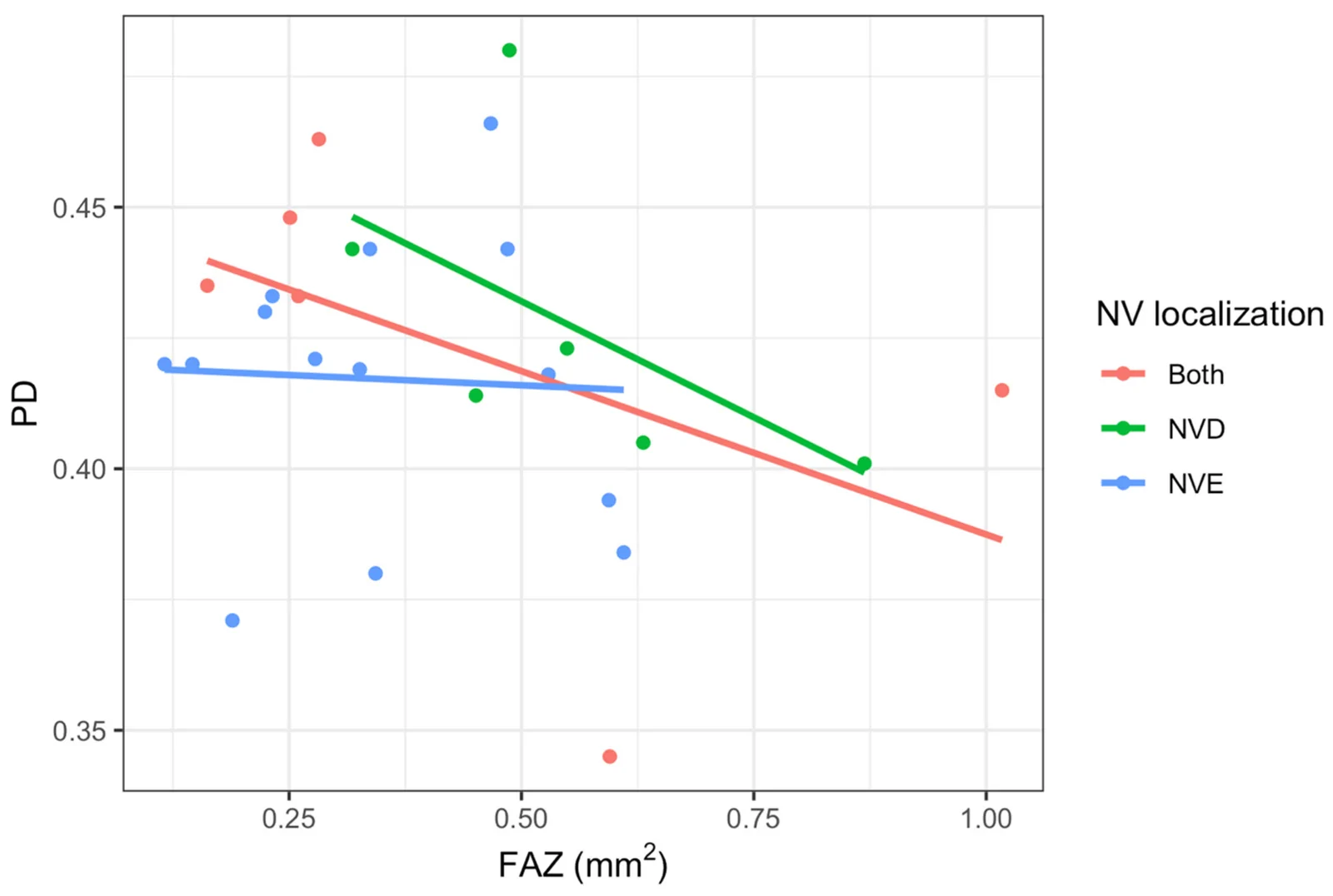
Regression analysis between FAZ area and perfusion density (PD) in the whole retina slab. The green line showed the NVD group slope, the blue line NVE group and the red line the NVD + NVE group slope.

The regression analysis showed a linear relationship between FAZ area and PD in the whole retina slab (Figure 4). In the NVD group, PD decreased by −0.06 [confidence interval CI −0.23–0.09] (R^2^ = 0.05) (*p* = 0.31) at each mm^2^ of FAZ as well as in the NVD + NVE group in which the decrease was −0.02 [CI −0.15–0.10] (R^2^ = 0.14) (*p* = 0.58). This regression analysis for the NVE showed a slight positive relationship with an increase of 0.003 [CI −0.08–0.09] (R^2^ = 0.08) (*p* = 0.31) of the PD each mm^2^ of FAZ.

**Figure 5:**
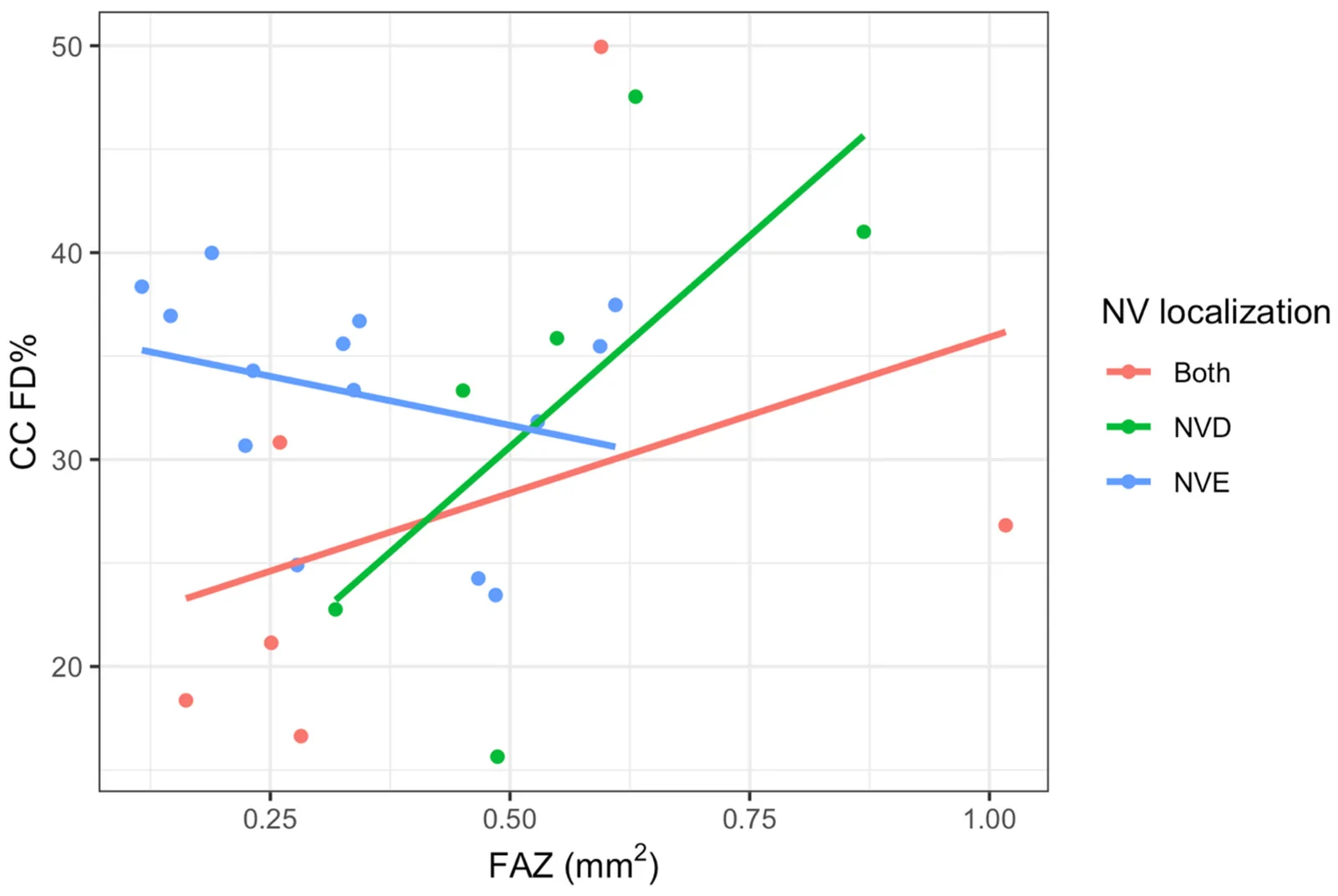
Regression analysis between FAZ area and choriocapillaris (CC) flow deficit. The green line showed the NVD group slope, the blue line NVE group and the red line the NVD + NVE group slope.

The regression analysis showed a linear relationship between FAZ and CC FD (Figure 5). In the NVD group, CC FD% increased by 40.75% [CI −25.24–106.73] (R^2^ = 0.27) (*p* = 0.16) at each mm^2^ of FAZ in NVD group as well as in the NVD + NVE group by 15.07% [CI −33.24–63.38] (R^2^ = 0.05) (*p* = 0.43). This regression analysis for the NVE showed a negative relationship with a decrease of −9.46% [CI −29.37–10.44] (R^2^ = 0.005) (*p* = 0.32) of the CC FD each mm^2^ of FAZ. The low R^2^ values for both regression analyses reflect the high data variability associated with our sample size.

## 4. Discussion

In this pilot study we explored macular vascular assessment and structural parameters of PDR eyes based on the NVs location. The topographical distribution of NVs, seemed express different structural and vascular macular profile.

On structural parameters, in our cohort, we analyzed CMT values distinguishing between NVD, NVE and NVD + NVE and no difference emerged between groups. Previous morphological analysis focused on CMT, did not find any significant differences in CMT measurement considering different degree of DR [26]. In our cohort we included only patients without CS-DME in order to exclude the bias due to the presence of macular edema on the volume examination, layer segmentation and vessel displacement, and no differences in CMT were found between groups.

Interestingly, a layer-by-layer analysis showed a thickening of RNFL, GCL and IPL in eyes with evidence of NVD compared to others. This thickening of inner retinal layers seemed quite peculiar and no previous studies have reported similar results on PDR, considering the topographical distribution of NVs. Previous studies exploring OCT characteristics of PDR eyes, were mostly focused on the differences with NPDR stages, highlighting the presence of several OCT biomarkers of a more severe DR stage, as disorganization of retinal inner layers (DRIL), MAs, hyperreflective foci (HRF), intraretinal cysts (IRC) and vitreo-retinal abnormalities in PDR [27]. It was also reported that patients with severe NPDR showed lower inner retinal layer thickness correlated with lower vessel density in SCP [28].

This peculiar thickening of the inner retinal layers that we have found in our sample might be differently interpreted, in fact might be possible related to a mechanical effect exerted by NVDs on the inner retinal surface. Owing to their topographic proximity to the macular region, these NVs of the disc may induce mechanical remodeling, leading to a thickening of the innermost retinal layers. On the other hand, this thickening could also be expression of a compensatory response in which the inner retinal thickens due to increased metabolic demand in response to a more pronounced ischemic insult. This hypothesis suggests that the presence of NVDs may reflect greater microvascular impairment, to which the retina responds by thickening as part of a neurodegenerative compensatory mechanism. However, while this finding is intriguing, it should be considered preliminary and interpreted with caution given the pilot nature of this study and the limited sample size.

In our study, we also found no differences in choroidal thicknesses between groups. Ghassemi et al. [29] reported data in a cohort of diabetic patients with different severity degree from normal, mild NPDR to PDR. The author demonstrated that the choroidal thickness showed a persistent thinning from normal to severe NPDR and then a thickening in patients developing PDR. Contrarily, Turkseven Kumral et al. [26] reported a significant difference in ChT between NPDR and PDR patients. A recent metanalysis evaluating the association between subfoveal ChT and DME reported no significance differences in PDR eyes with and without macular edema [30].

The analysis of OCTA parameters allows to identify a larger FAZ in NVD compared to other groups. Although the significance was marginal (*p* = 0.051), the FAZ area analysis revealed an interesting trend indicating a greater enlargement in case of NVD.

FAZ abnormalities were one of the first findings detected in DR in comparison to health controls [31], characterized by enlargement, worse circularity and larger perimeter [21,32]. Typically, the DCP was affected earlier than the SCP but both retinal slabs significantly worsen with diseases progression [33]. In our study FAZ was measured in the whole retina slab. This kind of measure is the most comprehensive in this advanced stage of the disease and, furthermore, FAZ size obtained using retinal vascular flow information for the entire retinal thickness, may be a more reliable measure of macular ischemia than FAZ size obtained for the segmented retinal vascular layers since the superficial and deep plexus connect around the FAZ [34].

Sodhi et al. [7] prospectively found a significant FAZ enlargement in PDR eyes showing NVD, and our results agree with those. Similar results were also reported by Elbendary et al. [35]. The FAZ enlargement represents a vascular impairment due to an ischemic damage [34,36] and, starting from our results, we can hypothesize that the presence of disc neovascularization might be expression of a more pronounced ischemic profile.

The regression analysis between FAZ enlargement and other OCTA vascular parameters showed that in patients presenting disc neovessels (in both, NVD and NVD + NVE groups), a reduction of PD and an increase of the CC FD% were found for each mm^2^ of FAZ enlargement. The low R^2^ values reflect the high data variability associated with our sample size, due to the small number of eyes analysed. Larger cohort studies will be necessary to confirm these observed trends.

Despite this limation, these findings could support the hypothesis of the existence of a great ischemic impairment in case of disc neovessels involving not only the retina but also the choroid.

In 2023 Nidhi et al. [1] analyzed the topographic distribution of NV and capillary nonperfusion (CNP) areas using UWF-FA. They found that diabetic neovascularization and CNP areas are distributed asymmetrically throughout the retina and, in particular, in patients with only NVD, the mean CNP area was higher in all four quadrants compared to the patients in whom only NVE was detected [1]. The authors concluded that the presence of NVD indicates a more advanced stage of PDR [1].

Furthermore, Shimizu et al. [8] reported a more extensive nonperfusion in NVD group than NVE supporting the hypothesis of our work of a more pronounced ischemic profile in PDR eyes with NVD.

In our study we used the PD analyzed in the whole retina, in the SCP and DCP respectively, to evaluate the retinal vascular assessment in the macular region and no significant differences were found in these metrics between groups. This finding may likely reflect the severe retinal impairment at this advanced stage of the disease and the inability to explore fine differences to distinguish between these groups; furthermore, detailed analyses will be needed to better describe whether any differences exist. We also explored the choroidal vascular involvement by means CC FD in the 6 × 6 mm macular slab and no significant differences between groups were found.

Choi et al. [37] described regions of CC flow impairment in patients with PDR but, interestingly even in diabetic eyes without retinopathy, a CC flow impairment could be measured. This was in line with histopathological evidence of choroidal changes in diabetic eyes, expressing the vascular insufficiency characterizing the diabetic choroidopathy [38] that might also influence the progression of diabetic retinopathy [39].

Recently, Yang et al. [33] in a cross-sectional study on 498 eyes from 296 T2DM patients with different levels of DR, reported that increased CC flow voids density, FAZ area and decrease vascular perfusion was associated with more severe DR.

Despite the increasing evidences of both, retinal and choroidal vasculature impairment and the hypothesis of an interconnection between retinal and choroidal vascular supply, although it is not yet clear which damage originates first.

The results of our exploratory analysis, revealed that the FAZ enlargement showed a negative relationship with retinal PD and a positive relationship with CC FD%, respectively in eyes with NVD, potentially indicating the double retinal and choroidal damage in the advanced stages of the disease and, in particular, in presence of disc neovascularization. These data should be interpreted with caution due to the low R^2^ values, but they may offer promising new perspectives for future, more in-depth investigations.

Our data on the regression analysis in the NVE group, showed an opposite direction than NVD. The CC impairment in NVE group did not show the positive relationship of NVD, corroborating possible differences in the vascular assessment in different types of PDR. It was surprising that in case of NVE a negative relationship between CC flow deficit and FAZ area was found, and we hypothesized that this behavior might be expression of a possible rearrangement of choriocapillaris vessels to compensate the ischemic injury, not possible in the NVD group due to a more advanced damage. Further studies with larger sample and the analysis of additional parameters could be useful to better understand this result.

This study presented several limitations as the retrospective nature and the small sample size. Another important limitation was the absence of clinical records regarding disease duration or glycaemic control, as the study focused solely on the stage of retinal disease and its structural features. In order to obtain a homogeneous analysis, we included only patients with a complete imaging with good quality images, reducing the sample and in particular, we excluded all CS-DME eyes to reduce the bias, obtaining more generalized results.

In conclusion our data support the hypothesis of a more pronounced ischemic damage in PDR eyes in presence of disc neovascularization, by means of structural and vascular assessment evaluated using OCT and OCTA metrics. A layer-by-layer analysis showed a peculiar thickening of inner retinal layers in case of NVD and also a FAZ enlargement results of ischemic injury. Further studies with a larger sample and additional analysis might be useful to endorse the result of this pilot study.

## Figures and Tables

**Table 1:** Demographic and imaging characteristics of study population.

	Overall	NVD Group	NVE Group	NVD + NVE Group
n. of patients	26	6	14	6
	mean ± SD	mean ± SD	mean ± SD	mean ± SD
Age (years)	53.3 ± 14.2	45.2 ± 15.2	55.5 ± 14	56.3 ± 12.8
CMT (μm)	311 ± 39.4	321 ± 38.8	297.5 ± 39.2	331.8 ± 33.1
ChT (μm)	288.4 ± 68.4	275 ± 95.1	298.5 ± 53.1	278.1 ± 79.6
FAZ area (mm^2^)	0.41 ± 0.22	0.55 ± 0.18	0.34 ± 0.16	0.42 ± 0.32
PD whole retina	0.43 ± 0.02	0.43 ± 0.02	0.43 ± 0.02	0.43 ± 0.03
PD SCP	0.42 ± 0.03	0.42 ± 0.03	0.41 ± 0.02	0.42 ± 0.04
PD DCP	0.41 ± 0.03	0.40 ± 0.03	0.41 ± 0.03	0.42 ± 0.03
CC FD (%)	31.66 ± 8.9	32.69 ± 11.74	33.09 ± 5.41	27.28 ± 12.3

NVD: disc neovessel, NVE: neovessel elsewhere, SD: standard deviation, CMT: central macular thickness, ChT: subfoveal choroidal thickness, FAZ: foveal avascular zone, PD: perfusion density, SCP: superficial capillary plexus, DCP: deep capillary plexus, CC FD: choriocapillaris flow deficit.

## Data Availability

All data generated or analyzed during this study are included in this article. Further enquiries can be directed to the corresponding author.
